# The Inhibitory Effect of Quercetin on Biofilm Formation of *Listeria monocytogenes* Mixed Culture and Repression of Virulence

**DOI:** 10.3390/antiox11091733

**Published:** 2022-08-31

**Authors:** Pantu Kumar Roy, Min Gyu Song, Shin Young Park

**Affiliations:** Institute of Marine Industry, Department of Seafood Science and Technology, Gyeongsang National University, Tongyeong 53064, Korea

**Keywords:** *Listeria monocytogenes*, quercetin, biofilm, stainless steel, rubber, hand gloves, food safety, gene expression

## Abstract

*Listeria monocytogenes* is the species of foodborne pathogenic bacteria that causes the infection listeriosis. The food production chain employs various methods to control biofilms, although none are completely successful. This study evaluates the effectiveness of quercetin as a food additive in reducing *L. monocytogenes* mixed cultures (ATCC19113, ATCC19117, and ATCC15313) biofilm formation on stainless steel (SS), silicon rubber (SR), and hand glove (HG) coupons, as well as tests its antimicrobial activities. With a minimum inhibitory concentration (MIC) of 250 µg/mL, the tested quercetin exhibited the lowest bactericidal action with no visible bacterial growth. In contrast, during various experiments in this work, the inhibitory efficacy of quercetin at sub-MICs levels (1/2, 1/4, and 1/8 MIC) against *L. monocytogenes* was examined. A control group was not added with quercetin. The current study also investigates the effect of quercetin on the expression of different genes engaged in motility (*flaA*, *fbp*), QS (*agrA*), and virulence (*hlyA*, *prfA*). Through increasing quercetin concentration, swarming and swimming motility, biofilm formation, and expression levels of target genes linked to flagella motility, virulence, and quorum-sensing were all dramatically reduced. Quercetin (0–125 μg/mL) was investigated on the SS, SR, and HG surfaces; the inhibitory effects were 0.39–2.07, 0.09–1.96 and 0.03–1.69 log CFU/cm^2^, respectively (*p* < 0.05). Field-emission scanning electron microscopy (FE-SEM) corroborated the findings because quercetin prevented the development of biofilms by severing cell-to-cell contacts and inducing cell lysis, which resulted in the loss of normal cell shape. Our findings suggest that plant-derived quercetin should be used as an antimicrobial agent in the food industry to control the development of *L. monocytogenes* biofilms. These outcomes suggest that bacterial targets are of interest for biofilm reduction, with alternative natural food agents in the food sector along the entire food production chain.

## 1. Introduction

A common foodborne bacteria called *Listeria monocytogenes* produces the disease listeriosis, which has a 20–30% fatality rate in susceptible individuals [1]. Because of its high resilience, the presence of *L. monocytogenes* on food contact surfaces is especially problematic. Listeriosis is a deadly condition caused by consuming food contaminated with *L. monocytogenes*. Among foodborne zoonoses, this rare disease has the highest mortality rate, placing a heavy financial, social, and medical burden on society [2]. According to the Center for Disease Control and Prevention-2022 (CDC), approximate estimates of the number of cases per year are 1600, with 260 fatalities [3]. Pregnant women and their unborn children, seniors 65 years of age and older, and those with compromised immune systems are the groups most prone to contract the pathogen [3].

Foodborne illnesses are brought on by eating contaminated foods, and an increase in the prevalence of these diseases is a serious public health issue [4]. Multiple pathogen survival and colonization processes, including as biofilm formation and motility, are associated with pathogen infections. During the early stages of adhesion, motility is related to cell-surface attachment and the subsequent production of biofilms, and it helps bacteria withstand both the host’s immune system and antibacterial agents [5]. A foodborne bacterium called *L. monocytogenes* can grow biofilms on both biotic and abiotic surfaces, which helps it survive in conditions where food is processed [6]. Since non-motile *L. monocytogenes* mutants are incapable of creating biofilms, it is unclear what specific molecular foundation underlies its ability to create biofilms [7]. Nevertheless, flagellar motility is crucial. The virulence regulator gene *PrfA*, which is both a master transcriptional activator of other virulence genes and a potential mediator of biofilm creation, and the quorum-sensing (QS) system in *L. monocytogenes*, encoded by the accessory gene regulator (*agr*) locus—*agrBDCA*—both play critical roles in biofilm formation [8,9], besides showing that the *L. monocytogenes agrD* deletion mutant clearly impairs biofilm formation and identifying six biofilm-related genes in *L. monocytogenes*, including four flagella-related (motility genes) *flaA, fliP, fliG,* and *flgE*, and two agr QS system genes (*agrA* and *agrB*) [6,10].

Compared to planktonic cells, biofilms have a thousand times higher resistance for all antimicrobial treatments. The removal of biofilm with common antibiotics and cleaning agents is therefore challenging [11]. Microscopical examinations have demonstrated that the processes of biofilm development were initial adhesion, microcolony production, and biofilm maturation [12]. Aggressive chemicals, like sodium hydroxide or sodium hypochlorite, are frequently employed in the food sector to reduce the negative impacts of biofilms [13]. However, such methods might damage the environment by corroding equipment and materials [14,15]. Therefore, it is essential to make a successful strategy that can manage and eradicate bacterial biofilm. In comparison to free-living bacterial cells, bacterial growth that protects itself by embedding cells regularly in extracellular polymeric substances (EPS) is known as biofilms [16,17]. This increases the bacteria’s ability to survive acquaintance with antimicrobial agents [17,18]. Many biofilm-related genes regulate the continuous, dynamic processes that lead to the formation of biofilms, including cell attachment, EPS synthesis, resource capture, detachment, and dispersal. Cross contamination may be a significant source of human diseases, according to reports [19]. Biofilm is an important target for lowering contamination and infections brought on by *L. monocytogenes*.

Bacteria live in microbial biofilms, which shield them from physical harm, desiccation, and antibiotic agents [20]. According to numerous studies, foodborne pathogens persist as biofilms on food-contact surfaces (e.g., plastic, steel, glass, and rubber) and impact the quantity, quality, and safety of food products [1,6,21,22]. Additionally, they destroy surfaces and equipment, contaminate food on a constant basis, pose a significant risk to public health, and their control is a significant barrier in the food production chain [23]. To prevent foodborne infections, natural, plant-extracted antimicrobial compounds are typically regarded as secure, efficient, and environmentally friendly [24,25]. With a broad range of activities against numerous bacterial and fungal infections, convinced plant extracts have long been extensively used for food safeguards and disease prevention [17,25,26]. An alternate remedy for *L. monocytogenes* biofilms is derived from plants [27,28]. In particular, phenolic compounds prevent this pathogen from forming biofilms through a variety of mechanisms of action; for example, treatment with gallic acid (at 5.8 mM) decreased *L. monocytogenes* motility, changed the physicochemical characteristics of the cell surface, and decreased its metabolic activity [29]. The prevention of biofilm on polystyrene surfaces was linked to these changes. Similar to this, resveratrol (0.2–0.8 mM) lowered the metabolic activity of the adherent cells and decreased the total biomass of *L. monocytogenes* biofilms [30].

Use of inhibiting substances that interfere with the quorum sensing (QS) is one of the preventative measure for enhancing food quality and safety [31,32]. One of them targets QS, a mechanism that allows cells to communicate with one another and allows germs to survive under adverse conditions [33,34]. When bacterial concentrations approach a predetermined concentration threshold, signaling molecules or auto-inducers are secreted, which control the expression of virulence genes at bacterial densities [34]. Numerous virulence factors, such as the production of nuclease, hemolysin, lipase, protease, and prodigiosin, as well as the development of biofilms and motility, are regulated by QS [33,34]. QS in many bacteria can be disrupted by phenolic chemicals generated from plants [31]. Plant compounds are an alternative control method against *L. monocytogenes* biofilms, and one of the most investigated flavonoid molecules having functional characteristics in this context is quercetin. Flavonoids have become well known for having anti-inflammatory, antioxidant, antibacterial, and anticancer properties [35,36], in addition to their potential QS system inhibitory properties [36,37,38]. Many fruits and vegetables, including apples, onions, red grapes, berries, tomatoes, and tea, contain quercetin, a flavonoid-based compound [39]. Due to its anti-inflammatory, anticancer, and neuroprotective properties, it has a wide range of applications [40,41,42]. Owing to its three-ring structure with five hydroxyl groups, it possesses especially strong antioxidant capabilities [22,35,40,43]. Antioxidants can reduce oxidative stress and prevent biofilm formation by eliminating reactive oxygen species (ROS) accumulated in bacterial cells [22,35,43]. As a result, antioxidants are potent antibiofilm agents [40,44]. One of the primary processes by which oxidative stress induces bacteria to develop biofilms is as a survival strategy. Additionally, it has already been demonstrated that quercetin has antibacterial properties against both Gram-positive and Gram-negative bacteria [39], including *Staphylococcus aureus* [39,45], *Escherichia coli* [39,46], and *Pseudomonas aeruginosa* [39,47]. Furthermore, quercetin may also inhibit *L. monocytogenes* biofilms. Since the antibiofilm effect is seen at non-lethal concentrations, it is likely that this substance blocks particular biofilm formation pathways, such as initial adhesion or EPS synthesis. According to studies, the phenolic acids, gallic, ferulic, and caffeic acids, as well as the flavonoids rutin and catechin, interfere with the *L. monocytogenes’* ability to adhere to stainless steel surfaces [48]. This result might be attributed to their ability to reduce bacterial movement and alter the physicochemical characteristics of different substrates (e.g., surface charge and hydrophobicity). Consequently, quercetin affects the ability of foodborne pathogens to regulate biofilms in the food industry. We hypothesize that quercetin might impact *L. monocytogenes* biofilm development because flavonoids have altered the pathogenicity of this pathogen.

Therefore, in the current investigation, we aimed to evaluate the effects of quercetin at sub-MIC for its ability to control *L. monocytogenes* mixed-cultures biofilm formation on food-contact surfaces, as well as flagella motility, virulence, and QS-gene expression in relative levels.

## 2. Materials and Methods

### 2.1. Bacterial Strain Culture and Growth Conditions

*Listeria monocytogenes* was obtained from American Type Culture Collection (Manassas, VA, USA), with strains ATCC19113, ATCC19117, and ATCC15313, and used for the biofilm forming assays. The bacteria were cultured in tryptic soy broth (TSB, BD Difco, Franklin Lakes, NJ, USA) at 30 °C for 24 h, followed by another sub-culture at 18 h [49]. The cultures were centrifuged (11,000× *g* for 10 min) and washed two times with phosphate-buffered saline (PBS; Oxoid, Basigstoke, Winchester, UK). After that, peptone water (PW; Oxoid, Basingstoke, Winchester, UK) was added to the final bacterial solution to dilute it until it contained 10^5^ log CFU/mL of bacteria. Then, the strains were mixed together to make a mixed culture suspension for further experiments. Following a 48 h-incubation period at 30 °C, the final concentration was ascertained using the spread plate method on PALCAM agar (Oxoid, Basingstoke, Winchester, UK) using a PALCAM-selective supplement.

### 2.2. Preparation for Food-Contact Surfaces

With few modifications, sample preparation was done as explained in our earlier investigations [49]. Using a sterile scissors, latex hand gloves (HG, Komax Industrial Co., Ltd., Seoul, Korea) were cut into 2 × 2 cm^2^ coupons, and silicon rubber and stainless-steel coupons (2 cm × 2 cm × 0.1 cm) were also used. Following the removal of any dirt, the coupons were cleaned with sterile, distilled water (DW). The coupons were sterilized by autoclaving (121 °C for 15 min) and dried overnight [49]. The coupons were completely submerged into 50 mL Falcon tubes containing 10 mL TSB, inoculated with final concentrations (100 µL) of bacteria (10^5^ CFU/mL), and then incubated for 24 h at 30 °C without shaking for further experiment.

### 2.3. Quercetin Preparation and Determination of Minimum Inhibitory Concentration (MIC)

From Sigma-Aldrich, we obtained quercetin (Q-4951) (St. Louis, MO, USA). After being dissolved in dimethyl sulfoxide (DMSO, Sigma-Aldrich, St. Louis, MO, USA), the product was used to make a stock solution with a concentration of 1 mg/mL. The MIC was verified and very slightly modified from a previous study [22]. A twofold serial dilution approach using TSB was used to establish the MIC of quercetin against *L. monocytogenes* mixed cultures. A total of 100 µL of quercetin, serially diluted with TSB and 100 µL of bacterial suspension (10^5^ log CFU/mL), were combined in 96-well plates (Corning Incorporated, Corning, Inc., Corning, NY, USA). Each well had a total amount of 200 µL. A microplate reader (Spectra Max 190, Sunnyvale, CA, USA) was used to measure absorbance (600 nm) while the plates were kept in a 30 °C incubator for 24 h. After an overnight incubation at 30 °C, aliquots (100 µL) taken from the wells that had no discernible growth were plated on PALCAM agar plates and the number of colonies were counted. Triplicates of this experiment were conducted.

### 2.4. Analysis of Motility

Motility experiments were carried out in this study with minor variations from those previously published [22]. This test was conducted to verify the effect of quercetin on the two forms of the *L. monocytogenes* mixed-cultures motility (swimming and swarming). Bacto agar (BD Dicfo, Franklin Lakes, NJ, USA) was mixed with TSB at rates of 0.3% and 0.5% to provide the media for the swimming and swarming studies, respectively. Each plate was filled with the autoclaved medium. Quercetin was added and thoroughly mixed in before it was set.

### 2.5. Biofilm Formation and Detachment Process

With slight adjustments, the procedure was carried out as described previously [22]. The MIC in this study was 250 µg/mL, and the inhibiting effect of biofilm was seen at sub-MIC levels. Control, 1/8, 1/4, and 1/2 MIC concentrations were used in this study. The prepared samples were placed in a 50 mL conical tube, with 10 mL TSB, quercetin and 100 µL of bacterial suspension (10^5^ log CFU/mL). They were then thoroughly combined with a vortex mixer (Scientific Industries, SI-0256, Bohemia, NY, USA) before being incubated for 24 h at 37 °C. To get rid of bacteria that had somewhat attached to the surfaces after the biofilm formation, the coupons were washed twice with distilled water (DW) [22,35]. After being thoroughly washed, the coupons were placed in 10 mL of peptone water (PW; BD Diagnostics, Franklin Lakes, NJ, USA)), which contained 10 glass beads that had been sterilized [22,50]. This bacterial sample was serially diluted before being placed into PALCAM agar plates as an inoculum. The colonies on the plates were counted after 24 h in a 30 °C incubator. The inhibition values were calculated by subtracting the populations of each concentration (0, 1/8, 1/4, and 1/2 MIC) from the populations of each group.

### 2.6. Field Emission Scanning Electron Microscopy (FE-SEM)

To confirm the biofilm inhibition by quercetin (Control, 1/4, and 1/2 MIC) on food-contact surfaces (HG and SR), the surfaces were observed by FE-SEM. With minor modifications, samples were prepared according to a previous study [22]. The samples were briefly fixed with 2.5% glutaraldehyde in PBS and stored at room temperature for 4 h. After that, it was treated with ethanol (50, 60, 70, 80, and 90% for 15 min serially) 100% for 15 min two times. Then, the samples were dehydrated by soaking (33, 50, 66, and 100% hexamethyldisilazane in ethanol) for 15 min serially. The samples were dried in a fume hood for 3 h and platinum sputter-coated (Q150T Plus, Quorum, UK) and observed by FE-SEM (Hitachi/Baltec, Tokyo, Japan, S-4700) [6].

### 2.7. RNA Extraction, cDNA Synthesis and Real-Time PCR (RT-PCR)

With a few minor adjustments, the experiment was carried out as previously described [22]. RT-PCR was carried out to verify the effects of quercetin on the expression of virulence, flagella motility, and the quorum-sensing gene. Each Falcon^®^ tube containing 10 mL TSB with quercetin received an inoculation of the bacteria (10^5^ log CFU/mL). They were kept in an incubator at 30 °C for 24 h. Total RNA was collected using the RNeasy Mini kit (Qiagen, Hilden, German), followed by the manufacturing protocol. All samples of RNA were normalized as a concentration of 0.5 µg/µL. Using a Maxime RT PreMix (Random Primer) kit (iNtRON Biotechnology Co., Ltd., Seoul, Gyeonggi-do, Korea), complementary DNA (cDNA) was produced after the RNA yield and the purity was assessed using a spectrophotometer at 260/280 nm and 260/230 nm (NanoDrop, Bio-Tek Instruments, Chicago, IL, USA) [51]. Table 1 lists the primers.

The housekeeping gene was 16S rRNA. In a total volume of 20 µL, the cDNA sample was combined with the 10 pmol/μL primers (F and R) and the Power SYBR Green PCR Master Mix (Applied Biosystems, Thermo Fisher Scientific, Warrington, UK). A CFX Real-Time PCR System (Bio-Rad, Hercules, CA, USA) was used to perform the RT-PCR analysis. Utilizing the 2× Real-Time PCR Master Mix and 1 µL of cDNA as a template, RT-qPCR was carried out. A CFX Real-Time PCR System was used to conduct the real-time PCR. Initial denaturation for the PCR reaction took place at 95, 50, and 72 °C for 20 s each [52,53,54]. The expression levels of the target genes were standardized and normalized in relation to the critical threshold (CT) mean values of *rpIU* as a housekeeping gene by the 2^−^^△△Ct^ method analysis [55,56,57].

### 2.8. Statistical Analysis

Each of the experiments were performed at least three times. All data were expressed as a mean ± standard error of mean (SEM). Statistical significance was set at *p* < 0.05 when Ducan’s multiple-range test (Tukey: compare all pairs) and one-way ANOVA were performed using SAS software version 9.2 (SAS Institute Inc., Cary, NC, USA) to determine the significance.

## 3. Results

### 3.1. Determination of MIC

Quercetin is dose dependent from species to species. The MIC was established as the lowest quantity, with no visible bacterial growth. The outcomes indicated that the MICs of quercetin against *L. monocytogenes* mixed cultures (ATCC19113, ATCC19117, and ATCC15313) was 250 µg/mL. For further experiments in this study at different sub-MICs, 125, 62.5, and 31.25 µg/mL of quercetin were used.

### 3.2. Swimming and Swarming Motility Assays

**Figure 1:**
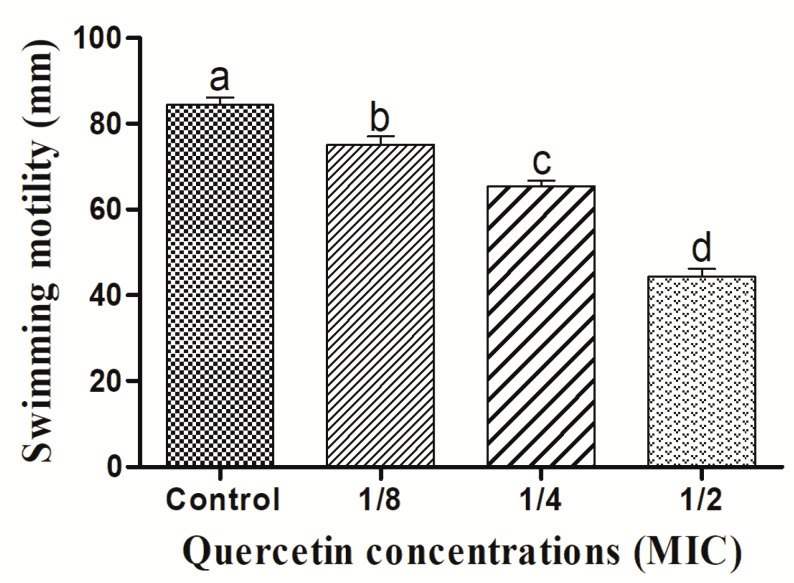
Swimming motility assay for *Listeria monocytogenes* mixed culture (ATCC19113, ATCC19117, and ATCC15313), with sub-MICs of quercetin (μg/mL) (control, 1/8, 1/4, and 1/2 MIC). Data represents the mean ± SEM of three independent replicates. a–d Values with different letters are significantly different by Duncan’s multiple-range test (*p* < 0.05).

**Figure 2:**
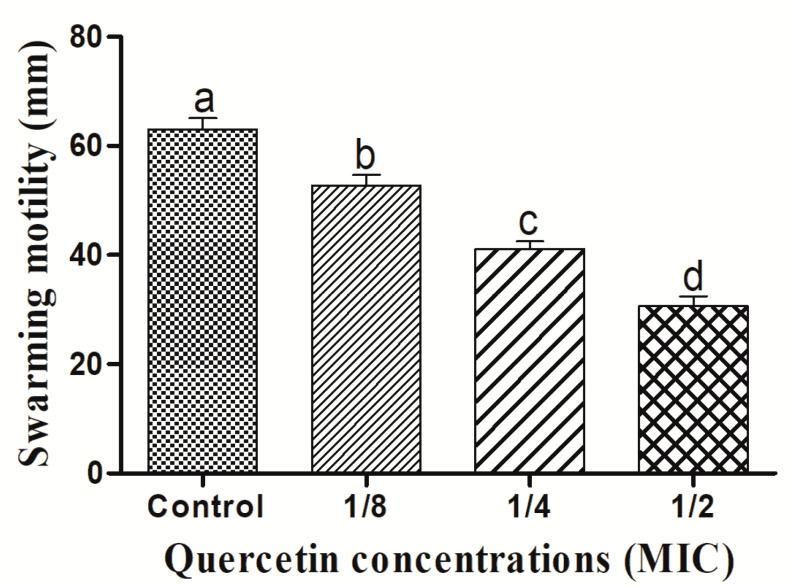
Swarming motility assay for *Listeria monocytogenes* mixed culture (ATCC19113, ATCC19117, and ATCC15313), with sub-MICs of quercetin (μg/mL) (control, 1/8, 1/4, and 1/2 MIC). Data represents the mean ± SEM of three independent replicates. a–d Values with different letters are significantly different by Duncan’s multiple-range test (*p* < 0.05).

For the formation of biofilms, bacterial flagella must be motile. *L. monocytogenes* mixed-culture flagella can be verified by swimming and swarming assays. The impact of quercetin on inhibiting *L. monocytogenes* mixed-culture motility is depicted in Figure 1 and Figure 2. Quercetin reduced *L. monocytogenes* mixed-culture motility by 17 and 77%, respectively, in the swimming experiment compared with the control group at 1/8 and 1/2 MIC.

Figure 2 depicts the quercetin’s inhibition of *L. monocytogenes* mixed-culture swarming motility. Quercetin thereby reduced *L. monocytogenes* mixed-culture motility by 16 and 58% at 1/8 and 1/2 MIC, respectively. Thus, in this experiment, as the quercetin concentration increased, swimming and swarming motility became more inhibited. Particularly in comparison to the control group, motility was significantly different, with 1/8, 1/4, and 1/2 MIC of quercetin.

### 3.3. Eradication Effect of Food Additive Quercetin on Food-Contact Surfaces against L. monocytogenes Mixed Culture

**Figure 3:**
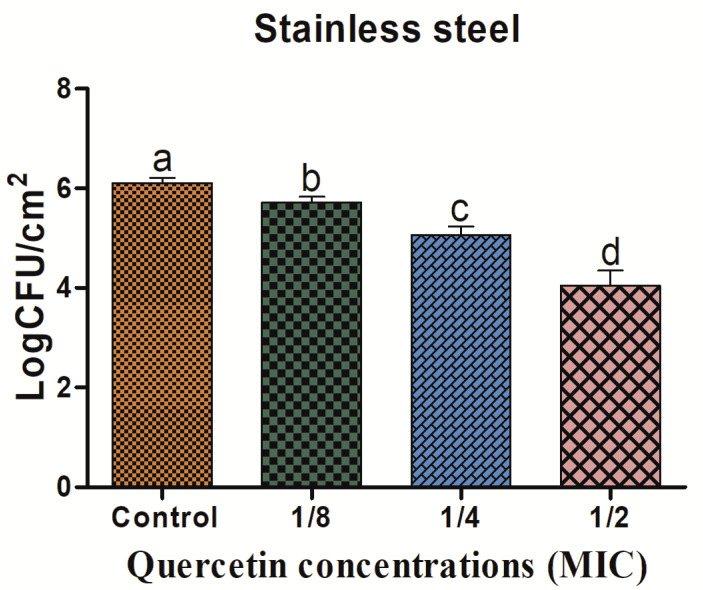
Inhibition of *Listeria monocytogenes* mixed culture (ATCC19113, ATCC19117, and ATCC15313) biofilm formation (24 h) on stainless steel surfaces by sub-MICs of quercetin (μg/mL) (control, 1/8, 1/4, and 1/2 MIC). Data represents the mean ± SEM of three independent replicates. a–d Values with different letters are significantly different by Duncan’s multiple-range test (*p* < 0.05).

The *L. monocytogenes* mixed-culture biofilm on the SS coupon shown in Figure 3 was inhibited by quercetin. As the quercetin concentration increased, the biofilm-inhibiting impact grew as well. The *L. monocytogenes* mixed-culture biofilm inhibition values on the SS surfaces were 0.39, 1.05, and 2.07 log CFU/cm^2^, respectively, at quercetin quantities of 1/8, 1/4, and 1/2 MIC.

**Figure 4:**
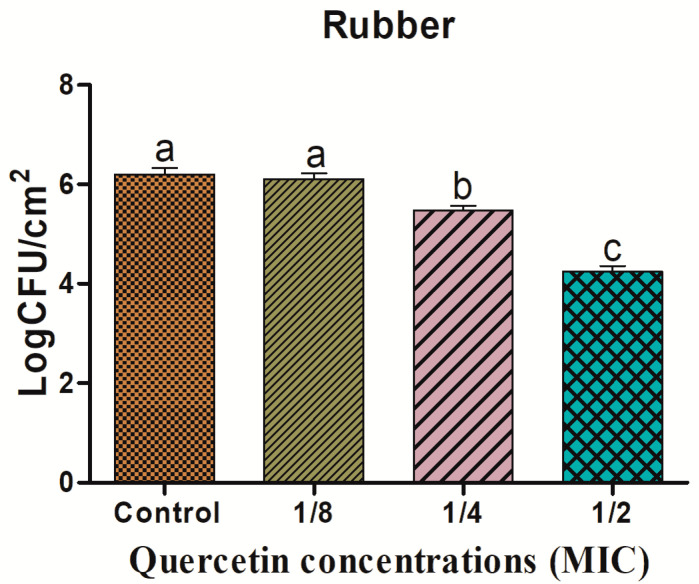
Inhibition of *Listeria monocytogenes* mixed culture (ATCC19113, ATCC19117, and ATCC15313) biofilm formation (24 h) on silicon rubber surfaces by sub-MICs of quercetin (μg/mL) (control, 1/8, 1/4, and 1/2 MIC). Data represents the mean ± SEM of three independent replicates. a–c Values with different letters are significantly different by Duncan’s multiple-range test (*p* < 0.05).

**Figure 5:**
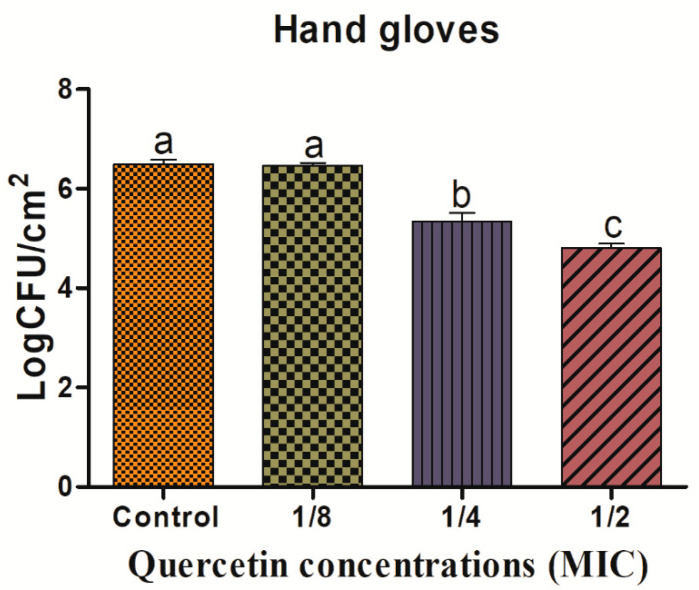
Inhibition of *Listeria monocytogenes* mixed culture (ATCC19113, ATCC19117, and ATCC15313) biofilm formation (24 h) on hand glove surfaces by sub-MICs of quercetin (μg/mL) (control, 1/8, 1/4, and 1/2 MIC). Data represents the mean ± SEM of three independent replicates. a–c Values with different letters are significantly different by Duncan’s multiple-range test (*p* < 0.05).

Biofilm reduction on SR surfaces is shown in Figure 4. The reduction of biofilms was 0.09, 0.73, and 1.96 log CFU/cm^2^, at 1/8, 1/4, and 1/2 MIC of quercetin concentrations, respectively. On the HG surface, the *L. monocytogenes* mixed-culture biofilm shown in Figure 5 was inhibited by quercetin. The *L. monocytogenes* mixed-culture biofilm inhibitory values were 0.26, 1.40, and 2.31 log CFU/cm^2^, at 1/8, 1/4, and 1/2 MIC of quercetin, respectively. Compared with the control group and other MIC groups, 1/4 and 1/2 MIC significantly inhibited biofilm formation (*p* < 0.05).

### 3.4. Visual Confirmation of Biofilm Reduction by Quercetin under FE-SEM

**Figure 6:**
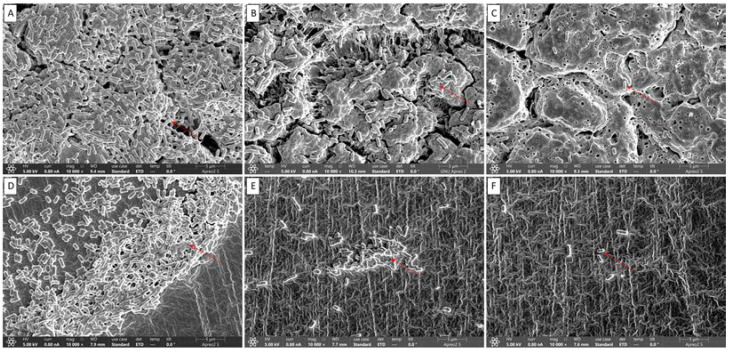
Representative scanning electron micrographs of *Listeria monocytogenes* mixed culture (ATCC19113, ATCC19117, and ATCC15313) biofilms formation in the presence of sub-MICs of quercetin on the hand glove surfaces: (**A**) control (0% quercetin); (**B**) 1/4 MIC; and (**C**) 1/2 MIC; and silicon rubber surfaces: (**D**) control (0% quercetin); (**E**) 1/4 MIC; and (**F**) 1/2 MIC.

The visual confirmation of biofilm inhibition by quercetin is shown in Figure 6. The biofilms were architecturally structured, with intact cell-to-cell contacts in control samples. Smooth and regular cells with intact cell membranes were observed in both the control (Figure 6A,D) and the quercetin-supplemented groups (Figure 6B,C,E,F). The rough and uneven appearance of the quercetin-treated bacterial cells indicated that the cells had lost their usual shape (Figure 6C,F). The cells lost their usual shape due to surface attachment on the rubber surface (Figure 6D–F). The red color mark indicates the attachment of biofilm cells in both the control (Figure 6A,D) and single groups and the lysis of biofilms in quercetin-treated samples (Figure 6B,C,E,F).

### 3.5. Motility, Biofilm Forming, Virulence and QS Sensing Relative Gene Expression Pattern

**Figure 7:**
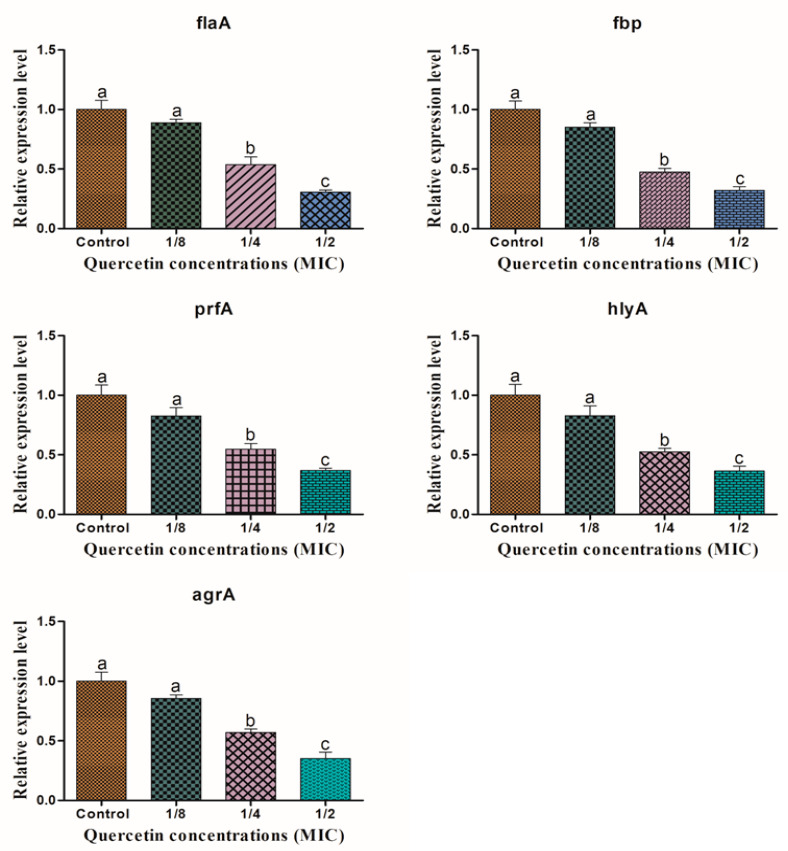
Relative expression levels of *flaA, fbp, prfA*, *hlyA,* and *agrA* genes in *Listeria monocytogenes* mixed culture (ATCC19113, ATCC19117, and ATCC15313), supplemented with sub-MICs of quercetin (control, 1/8, 1/4, and 1/2 MIC). a–c Different superscript letters indicate significant differences (*p* < 0.05) with three independent replicates.

Figure 7 shows the relative gene expression of *L. monocytogenes* mixed-culture motility, biofilm forming, virulence, and QS factor, as determined by RT-PCR in the sub-MICs of quercetin (from 0 to 125 µg/mL). At the various sub-MIC concentrations of quercetin, gene expression was considerably downregulated (*p* < 0.05), except 1/8 MIC, compared to the other groups.

## 4. Discussion

Natural compounds produced from plants offer a potentially helpful way to get beyond bacterial biofilm inhibitory mechanisms and regain the effectiveness of quercetin. Plant extracts, rather than being food additives, could be thought of as food constituents because they include quercetin. Unspecific protein kinase enzyme inhibitors include quercetin. In 2010, the FDA authorized the use of high-purity quercetin at levels up to 500 milligrams (mg) as an ingredient in many specific food categories [22]. The goal of the current investigation was to determine whether quercetin at sub-MIC levels could be used to inhibit the growth of *L. monocytogenes* mixed culture. Against *L. monocytogenes* mixed culture, quercetin has antibacterial efficacy, which we described in our study. We revealed that there was a dose-dependent bactericidal effect of quercetin against *L. monocytogenes* mixed culture and a considerable biofilm formation inhibition caused by quercetin using various techniques, including bacterial motility and the growth of biofilm. Quercetin not only inhibited bacterial growth but also repressed the expression of the virulence, flagellar motility, and the QS gene in response to *L. monocytogenes* mixed culture.

The MIC of quercetin was determined at 80 µg/mL for *Pseudomonas aeruginosa* and *Klebsiella pneumoniae*, 120 µg/mL for *Chromobacterium violaceum*, 250 µg/mL for *Salmonella Typhimurium*, and 95 µg/mL for *Yersinia enterocolitica* [22,35,58]. By encouraging surface adhesion, swimming and swarming locomotion affects bacterial biofilm development. Our results clearly show that quercetin dramatically decreased the test pathogens’ flagella-mediated motility compared to the control group (Figure 1 and Figure 2). The outcomes are analogous to those reported by Damte et al. [59], who found that plant extracts can reduce *Pseudomonas* swarming motility by 71%. Niu and Gilbert also found that cinnamaldehyde reduced *E. coli* swarming by inhibiting the growth of biofilms [60]. Similarly, quercetin reduced the motility at swimming (77 and 76%) and swarming (55 and 54.5%) against *S. Typhimurium* [22,35]. As a result, quercetin seems to inhibit the ability of foodborne pathogens to attach to the surfaces, hence reducing the formation of biofilms. Additionally, bacterial motility, including swimming and swarming, is a key component of pathogenicity. Hence, quercetin significantly decreased the motility of the tested microorganisms.

One of the most crucial components of a foodborne bacteria pathogenicity is the production of biofilms. QS is the one of the key elements in the development of biofilm by pathogens [61]. Thus, disrupting the signal-mediated QS system may control the development of biofilms. This study’s findings demonstrated that quercetin effectively decreased the biofilm development in test pathogens at all tested concentrations. Our results agree with those previously reported [22,35], which claimed that compared to the control group, quercetin (125 µg/mL)-treated foodborne pathogens *S. Typhimurium* rarely form biofilms on food and food-contact surfaces. As previously reported [48], 0.2 mM of quercetin was used against *L. monocytogenes* biofilm formation, which was necessary for the observation of changes brought on by quercetin [22]. To rule out any interference from quercetin (0.2 mM) on the planktonic populations during the experiment, its impact on *L. monocytogenes* planktonic growth kinetics was also assessed [22]. Because planktonic cells in the bulk medium continuously deposit onto layers of attached cells throughout normal development, it is important to recognize their role in biofilm formation. The results showed that the flavonoid quercetin prevented the development of *L. monocytogenes* biofilms, suggesting that quercetin affects biofilm formation mechanisms other than cell division [22,48]. However, increasing quercetin levels at 0.2 and 0.4 mM significantly (*p* < 0.05) increased biofilm reduction at 1.96 and 3.21 Log10 CFU/cm^2^ of viable surface, respectively [22,48], and completely supported our results at sub-MIC of quercetin; the biofilm was more inhibited by quercetin on food-contact surfaces (SS, SR, and HG) compared to the control group (Figure 3, Figure 4 and Figure 5). Foodborne pathogens can attach to plastic surfaces and form a biofilm, making the use of plastic cutting boards and cooking raw foods extremely prone to cross-contamination [22,62,63]. Additionally, compared to glass and SS surfaces, which are hydrophilic materials, plastic is more likely to allow *Salmonella* germs to stick to them [22,35,64]. Therefore, it is crucial to avoid contaminating the plastic cutting boards used while preparing or processing food because this leads to listeriosis. Other authors looked at the efficacy of quercetin to inhibit the formation of biofilms in *Staphylococcus epidermidis* [40]. Quercetin inhibited the growth of biofilms in a concentration-dependent manner. Quercetin reduced the growth of *S. epidermidis* biofilm by 90.5 and 95.3% at 250 and 500 µg/mL concentrations, respectively [40].

Quercetin likely disrupts cell-to-cell interactions, which causes bacteria to lose their usual structure [22]. These intercellular linkages promote bacterial colonization and the development of well-organized biofilms. When these connections are broken, the biofilm’s cells become detached and are then readily removed by washing [50]. According to the FE-SEM images of *L. monocytogenes* mixed culture, quercetin disrupts cell-to-cell connections (Figure 6), which is consistent with previous findings [22,35].

Many genes are crucial for *L. monocytogenes* pathogenicity, biofilm formation, and physiological traits. We have investigated the *L. monocytogenes* mixed-culture gene expression profiles for *flaA, prfA, agrA, hlyA*, and *fbp* to evaluate the potency of quercetin. It is an emerging technique for preventing biofilm development, minimizing pathogenic infections, and maintaining food safety to prevent or limit the QS production. Oxidative stress develops when ROS build up occurs inside the cell [65]. By enhancing microbial population adaptation and survival protection, oxidative stress significantly contributes to the production of biofilms [44]. Not just in human cells, but also in microbes, ROS are crucial signaling molecules [35]. To keep a healthy redox cycle going and to encourage microbial adhesion, ROS can act as both intracellular and extracellular stimulants [22,44]. This will eventually result in the formation of biofilms. There may be an accumulation because of a disruption in the redox cycle [22]. By generating ROS within cells and weakening the membrane integrity of bacterial cells, the antioxidant quercetin prevents the formation of biofilms [41]. The process by which *L. monocytogenes* biofilms form is intricate and involves numerous elements (e.g., virulence, QS, environmental, and other regulators) [6,10]. We have used *L. monocytogenes* mixed-culture cells treated with quercetin for RT-PCR, evaluated the expression levels of the target genes linked to the initial flagella motility, pathogenicity, and QS, and investigated the key regulator genes to learn more about the inhibitory mechanism of quercetin in biofilm formation. According to RT-PCR studies, quercetin suppressed the expression of some target genes linked to the development of *L. monocytogenes* mixed culture biofilms (Figure 7). These target genes play crucial roles in the development of biofilms and included those involved in motility (*flaA, fbp*), pathogenicity (*hlyA, prfA*), and QS (*agrA*) [6,10]. Quercetin at sub-MICs (1/2 and 1/4 MIC) significantly reduced the virulence expression of the *flgA, fbp, hlyA, prfA*, and *agrA* genes compared with the control group (Figure 7). It is advantageous that a tiny amount of quercetin may inhibit biofilm growth by regulating the expression of specific genes because large amounts are needed to destroy the bacterial cells [6]. Additionally, *flgA, fbp, hlyA, prfA*, and *agrA* are biofilm-associated genes, and quercetin at a concentration of 1/2 MIC significantly reduced their expression compared with the other groups (Figure 7). As previously reported, the effects of quercetin on downregulating the expression of the virulence genes *sigB, prfA, actA, inlA*, and *inlC* were responsible for developing biofilm by quercetin [9,48]. The quercetin appeared to decrease *L. monocytogenes* metabolic activity based on the down-regulated expression of the target genes [9].

Quercetin is unlikely to penetrate cells and relate to intracellular goals or transcriptional regulators directly. Quercetin, according to our assumption, may relate to certain membrane proteins, which would subsequently activate the bacterial signaling structure and cause transcriptional alterations in gene downregulation. Quercetin is a polyhydroxy hydrolytic compound that may form potent complexes with various macromolecules, including microbial adhesins and cell membrane proteins. Through bacterial signaling mechanisms like two-component systems, the changes in the membrane may cause the bacterial cells to adjust, changing how their genes are expressed.

## 5. Conclusions

We showed that quercetin at sub-MIC revealed efficient antimicrobial and possibly anti-pathogenicity capabilities against *L. monocytogenes* mixed culture on food-contact surfaces. Quercetin also significantly reduced viable bacterial cells, severed cell-to-cell contacts, detached existing biofilms, and drastically downregulated the expression of genes associated with motility, virulence and QS. Quercetin may therefore be applied as a substitute method to manage the biofilm of *L. monocytogenes* in food systems and to lower the risk of foodborne illnesses conveyed by this pathogen.

## Figures and Tables

**Table 1:** Primer used for Real-Time PCR.

Target Genes	Primer Sequences (5′-3′)	Amplicon Size (bp)	Gene Accession Number
*16S rRNA*	F: GGAGCATGTGGTTTAATTCG R: CCAACTAAATGCTGGCAACT	199	CP016470.1
* flaA *	F: TGGTTCTACAGTTGCTGGTT R: TTTAGTTGCGATGGATTGGT	184	CP087264.1
*prfA*	F: CAATGGGATCCACAAGAATA R: AGCCTGCTCGCTAATGACTTtd>	186	CP093220.1
*agrA*	F: ATGAAGCAAGCGGAAGAAC R: ACGACCTGTGACAACGATAAA	239	CP076669.1
*hlyA*	F: GCAATTTCGAGCCTAACCTA R: ACTGCGTTGTTAACGTTTGA	188	CP093220.1
*fbp*	F: GCCTGGTCTAAACTGGATTT R: CGCCATAAAGAGCGATACTT	189	CP090057.1

## Data Availability

Data is contained within the article.
